# Disseminated Diaper Dermatitis With Concomitant Body Rash: A Case Report

**DOI:** 10.7759/cureus.87273

**Published:** 2025-07-04

**Authors:** Catherine F Alapatt, Eileen Condren, Tanya Kadrmas-Iannuzzi

**Affiliations:** 1 Dermatology, Rowan-Virtua School of Osteopathic Medicine, Stratford, USA; 2 Pediatrics, Rowan-Virtua School of Osteopathic Medicine, Stratford, USA

**Keywords:** dermatitis, diaper dermatitis, irritant contact dermatitis, pediatric dermatology, rash in children

## Abstract

Diaper dermatitis is a rash typically localized to the genital area and may have various etiologies, including irritant and allergic contact dermatitis. In most cases, it resolves on its own with minimal to no medical intervention, and when treatment is necessary, it usually responds promptly. Here, we present a prolonged case of diaper dermatitis accompanied by a nearly full-body rash that was resistant to initial treatment with steroids, antibiotics, and antifungals.

## Introduction

Diaper dermatitis, commonly referred to as diaper rash, is an inflammatory condition that affects the diaper area, particularly the buttocks, genitals, and thighs [1]. It can result from various etiologies that contribute to the constellation of symptoms classified under diaper dermatitis. The most frequent causes include irritant contact dermatitis, allergic contact dermatitis, and candidal infections [2-4]. In general, the management of diaper dermatitis is minimal, as most cases resolve with little to no medical intervention. Initial treatment typically involves removing irritants from the affected area, maintaining proper hygiene, and applying topical emollients [5]. While most cases improve within two to three days, some may persist for 10 days or longer and require medical treatment. Depending on the underlying cause, interventions may include low-potency corticosteroids or antifungal agents [6]. If the condition proves refractory to initial treatments, more serious underlying causes, such as nutritional deficiencies, HIV, or Langerhans cell histiocytosis, should be considered [7]. Although diaper dermatitis, particularly of candidal origin, can spread beyond the diaper area, it is uncommon for the rash to extend to the trunk, extremities, and face. Here, we present a case of diaper dermatitis accompanied by a truncal body rash that was unresponsive to both antifungal and steroid creams.

## Case presentation

**Figure 1:**
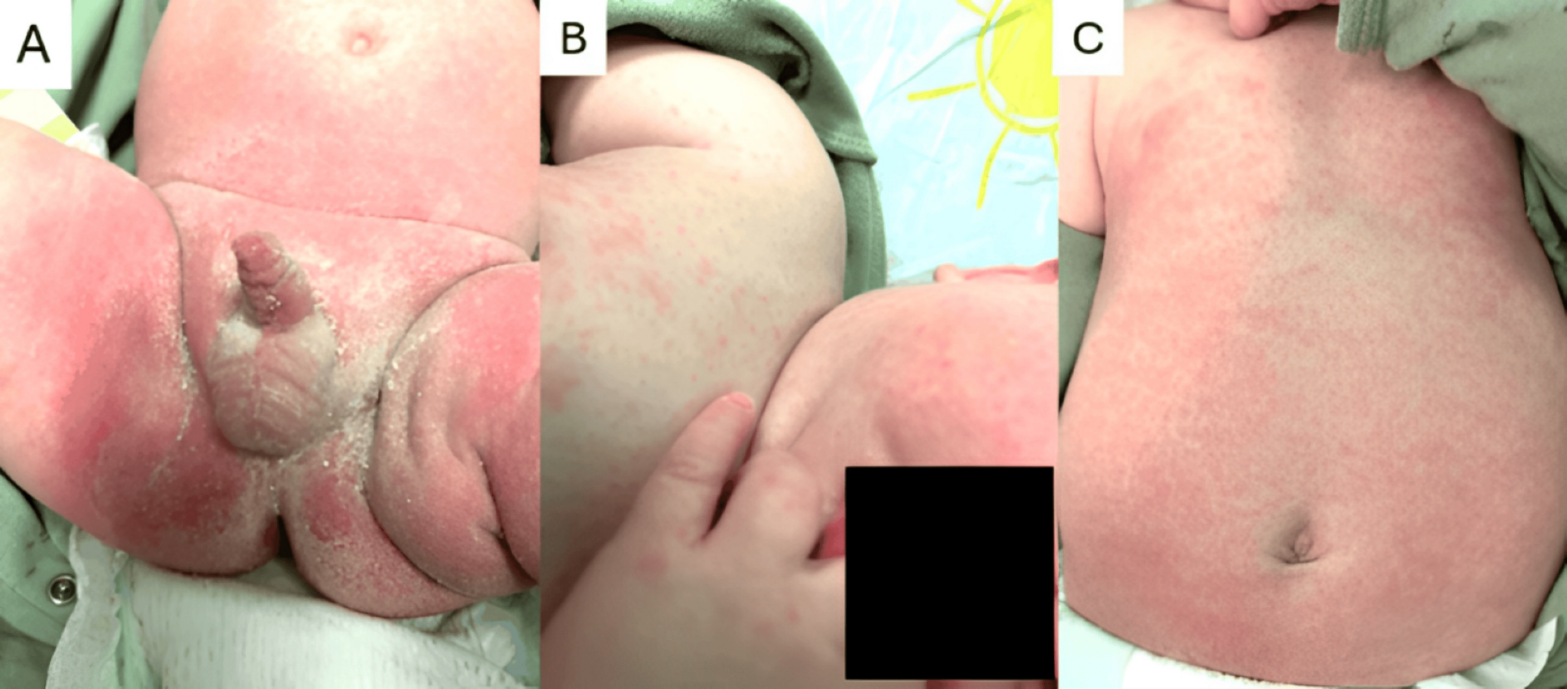
Initial presentation of diaper dermatitis (A) Genital area showing marked erythema, flaking, and scaling. (B) Maculopapular rash involving the face, hands, and chest, appearing on the third day. (C) Maculopapular rash on the trunk was also noted on the third day.

A six-month-old male with a history of redundant prepuce and phimosis presented to the office with a diaper rash that had begun five days earlier. The patient had experienced a similar-appearing diaper rash one month prior, which had resolved spontaneously without medical intervention. There were no preceding lesions, such as a herald patch, before the onset of the current diaper rash. According to the patient’s mother, a body rash developed on day 3 of the illness. An erythematous maculopapular rash was observed on the abdomen, chest, cheeks, arms, and hands (Figure 1).

The patient had no changes in activity and did not appear ill. He had no fever or cough. There were no known recent sick contacts, and he did not attend daycare. The patient was up to date on all vaccinations and had met all developmental milestones. His family had a history of sensitive skin and, accordingly, used fragrance-free detergents and sensitive-skin formulations for personal care products. However, there was no known family history of diagnosed skin conditions.

Despite the absence of systemic symptoms suggestive of infection, the rash appeared to be secondarily infected. This clinical diagnosis was based on the initial office presentation, which showed intense flaking, erythema, and skin fissures. During the physical exam, the patient scratched the affected area, displaying significant discomfort. Given the worsening diaper dermatitis and the appearance of a new body rash, a *Streptococcus *test was performed to evaluate for a possible candidal dermatitis secondarily infected with *Streptococcus*. A swab of the left buttock was also collected to assess for a different causative organism and to guide appropriate antibiotic therapy.

The patient was prescribed a 10-day course of cephalexin (2.4 mL of a 250 mg/5 mL suspension) to be taken three times daily, targeting a possible *Streptococcus *infection. In addition, nystatin cream was prescribed to be applied three times daily for presumptive candidal involvement. The family was instructed to return in four to five days if the rash persisted.

**Figure 2:**
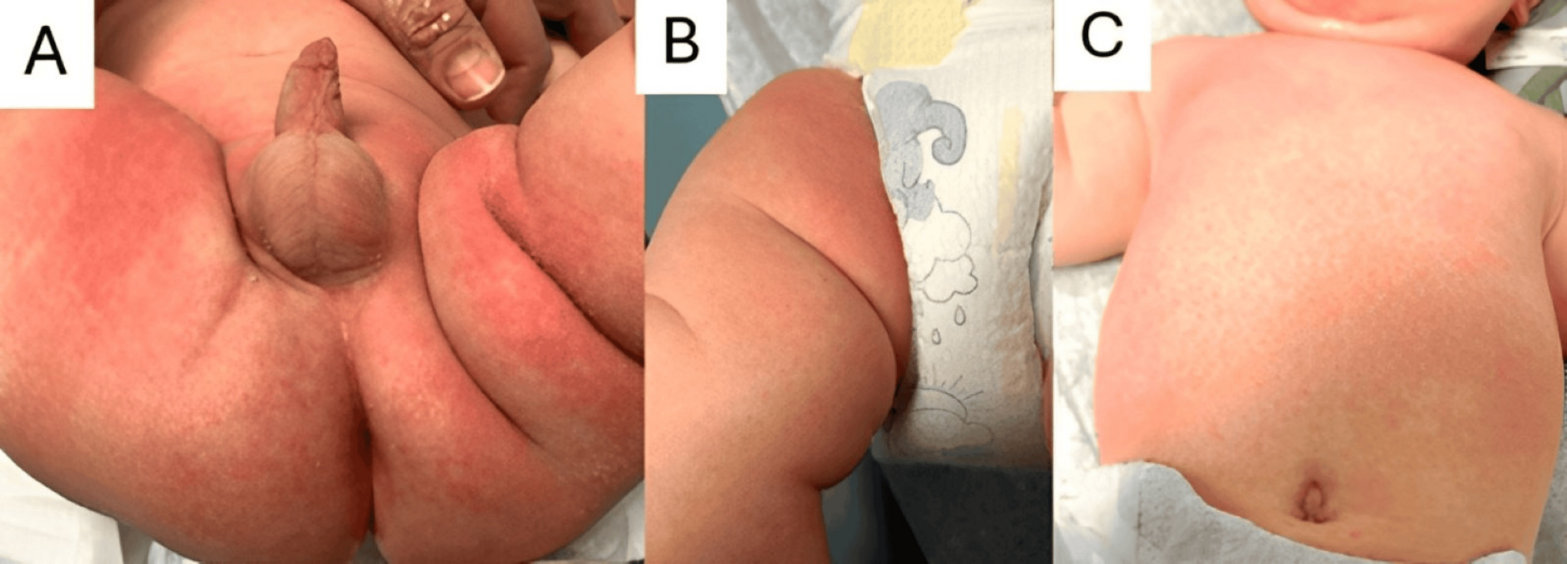
Follow-up presentation of diaper dermatitis (A) Genital area showing reduced erythema with resolution of flaking and scaling. (B) New maculopapular rash observed on the lower legs. (C) Persistent maculopapular rash on the trunk, continuing from the previous visit.

Both the rapid *Streptococcus *test and the Group A *Streptococcus *throat culture were negative. The skin swab from the left buttock, the primary site of the diaper rash, later identified *Citrobacter freundii*, *Raoultella planticola*, and *Enterococcus faecalis*. One week after the initial visit, the patient returned with a persistent rash (Figure 2). The diaper rash had moderately improved: it was less excoriated, and desquamation was no longer observed. However, erythema and pruritus persisted despite antimicrobial treatment. The body rash remained maculopapular and unchanged in appearance from the previous week, although its distribution had now extended to the legs.

During this visit, the patient did not appear ill, and the parents and grandparents denied any symptoms such as fever, chills, cough, or rhinorrhea. The patient was prescribed nystatin-triamcinolone cream to be applied to the diaper area twice daily for seven to 14 days.

## Discussion

Diaper dermatitis, although wide-ranging in etiology, is commonly classified into three well-known causes: irritant dermatitis, candidal dermatitis, and allergic contact dermatitis [8]. In cases of irritant dermatitis, it is hypothesized that proteolytic enzymes in stool and chemical irritants, in combination with excessive heat, moisture, and sweat retention, lead to irritation in the diaper area and ultimately cause dermatitis [6]. Candidal dermatitis, by contrast, is thought to result from colonization of the perineal and perianal regions by *Candida albicans*, leading to fungal infection [2]. These rashes typically present as beefy red plaques that may appear shiny and are usually more pronounced in the intertriginous folds [9]. They are not always limited to the diaper area and can present with satellite lesions, defined as areas of erythema bordered by red pustules [9].

For the treatment of irritant contact dermatitis, the “ABCDE” approach is typically employed. This includes airing out the affected area, applying a barrier cream, maintaining cleanliness, using appropriate diapers, and educating caregivers on treatment practices [10]. For diaper dermatitis involving candidal infection, a course of topical antifungals is generally sufficient, with nystatin being the drug of choice.

Although diaper dermatitis can involve satellite lesions, the presentation in this patient made it unlikely that the truncal rash was part of such a constellation. One possible explanation is that the patient had a resistant candidal dermatitis, which could account for only partial improvement with nystatin treatment. Reports have noted an increase in cases of candidal diaper dermatitis that are resistant to nystatin. In such cases, treatment should be switched to a topical azole [11]. While a nystatin-resistant candidal infection could explain the persistence of the diaper rash, it would not account for the development of a rash on other parts of the body.

Of note, *C. freundii *and *E. faecalis*, both cultured from the patient’s left buttock, are part of the normal intestinal flora. However, *R. planticola *is generally considered an environmental bacterium [12]. *Raoultella *species are rarely pathogenic and have only been implicated in human infections in a limited number of reported cases [13,14]. Given this patient’s atypical presentation - diaper dermatitis with a secondary, widespread maculopapular rash - it is possible that *R. planticola *triggered a diffuse dermatologic reaction. One case report describes a *Raoultella ornithinolytica *infection associated with a maculopapular rash in an otherwise healthy individual [14]. If *R. planticola *was the causative agent in this case, it could explain the poor response to both nystatin and oral cephalexin. Furthermore, the *R. planticola *isolate in this patient was not sensitive to first-generation cephalosporins.

Given the moderate improvement observed following treatment with nystatin and the family history of sensitive skin, it is possible that the patient had persistently dry, scaly skin as part of a variant of atopic dermatitis in an unusual distribution. Because atopic skin is prone to cracking and secondary infection, long-term management should include consistent skin moisturization and evaluation for potential environmental or food-related triggers. This case highlights the need for clinicians to be aware of the wide variability in diaper dermatitis presentations. While most cases are localized, this one involved an extensive rash beyond the diaper area, underscoring the importance of ruling out all potential infectious causes.

To our knowledge, no prior cases have reported diaper dermatitis presenting with a concomitant full-body rash. Although the exact etiology in this case remained uncertain, the application of general treatment principles still provided clinical benefit.

## Conclusions

Diaper dermatitis is a common condition encountered in pediatric dermatology. It rarely requires medical intervention, and when appropriate therapies are initiated, it typically resolves quickly. However, concomitant truncal rashes are exceedingly rare. When a patient presents with a rash that is resistant to standard treatment, as in the case described, it is essential to consider potential underlying causes. A thorough workup to identify secondary infections is critical in determining whether antibiotic therapy is warranted. If the dermatitis persists despite appropriate treatment, further evaluation for metabolic or immunologic disorders should be pursued. Additionally, distinguishing diaper dermatitis from atypical variants of atopic dermatitis remains a key diagnostic challenge. For patients with unusual or treatment-refractory presentations, we recommend a tailored approach that includes diagnostic testing to ensure comprehensive and effective care.
